# *In Silico* Molecular Docking and *In Vitro* Antidiabetic Studies of Dihydropyrimido[4,5-*a*]acridin-2-amines

**DOI:** 10.1155/2014/971569

**Published:** 2014-06-02

**Authors:** A. Bharathi, Selvaraj Mohana Roopan, C. S. Vasavi, Punnagai Munusami, G. A. Gayathri, M. Gayathri

**Affiliations:** ^1^Chemistry Research Laboratory, Organic Chemistry Division, School of Advanced Sciences, VIT University, Vellore, Tamil Nadu 632 014, India; ^2^Bioinformatics Division, School of Bio Sciences and Technology, VIT University, Vellore, Tamil Nadu 632 014, India; ^3^Division of Industrial Biotechnology, School of Bio Sciences and Technology, School of Advanced Sciences, VIT University, Vellore, Tamil Nadu 632 014, India

## Abstract

An *in vitro* antidiabetic activity on **α**-amylase and **α**–glucosidase activity of novel 10-chloro-4-(2-chlorophenyl)-12-phenyl-5,6-dihydropyrimido[4,5-*a*]acridin-2-amines (**3a**–**3f**) were evaluated. Structures of the synthesized molecules were studied by FT-IR, ^1^H NMR, ^13^C NMR, EI-MS, and single crystal X-ray structural analysis data. An *in silico* molecular docking was performed on synthesized molecules (**3a**–**3f**). Overall studies indicate that compound **3e** is a promising compound leading to the development of selective inhibition of **α**-amylase and **α**-glucosidase.

## 1. Introduction

**Figure 1:**
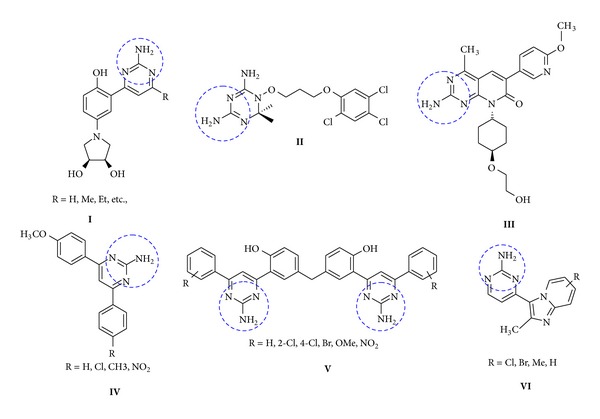
**I** CDK inhibitor,** II** kinase inhibitor,** III** antitumor,** IV** antimalarial,** V** antimicrobial, and** VI** anti-inflammatory.

Pyrimidine is a well-known biologically active nitrogen containing heterocyclic compound. In recent years researchers are much interested in the synthesis of pyrimidine analogues. Pyrimidine derivatives posse fungicidal [1], herbicidal [2], antidepressant [3], and antitumor properties [4, 5]. Synthetically prepared amino pyrimidine derivatives display a wide range of biological activities such as antibacterial [6], antitumor [7], and antiviral [8, 9]. Therefore, the substituted amino pyrimidine structure can be found in diverse clinically approved drugs. Interestingly, a substituted amino pyrimidine moiety was also suggested to account for the antioxidant activity [10]. Among those amino pyrimidine heterocycle, 2-aminopyrimidines have been widely used as pharmacophores for drug discovery. 2-Aminopyrimidines constitute a part of the DNA base pair molecules [11]. Compounds having potent anticancer activity, CDK inhibitory activity,** I** [12–14], antiproliferative activity [15], kinase inhibitor,** II** [16], antibacterial agents [17], antitumor,** III**, antidiabetic activity [18], antimalarial,** IV**, antiplasmodial agents [19], antimicrobial activity,** V** [20], and anti-inflammatory activity,** VI** [21] (Figure 1), contain amino pyrimidine moiety in the structure. The tricyclic, planar acridine moiety is responsible for intercalation between base pairs of double-stranded DNA through *π*-*π* interactions and, therefore, causes alteration in the cellular machinery [22]. Intercalative interaction of structurally related well-known intercalators, 9-aminoacridine (9AA), and proflavine (PF) was determined by means of fluorescence quenching study [23].

Due to numerous biological applications of 2-aminopyrimidine and acridine amine and its analogues, we have focused our research on synthesis of 2-aminopyrimidine by using pharmacologically important structural scaffold acridine pharmacophore.

Protein-ligand interaction is comparable to the lock-and-key principle, in which the lock encodes the protein and the key is grouped with the ligand. The major driving force for binding appears to be hydrophobic interaction [24].* In silico* techniques helps identifying drug target via bioinformatics tools. They can also be used to explore the target structures for possible active sites, generate candidate molecules, dock these molecules with the target, rank them according to their binding affinities, and further optimize the molecules to improve binding characteristics [25]. Diabetes mellitus (DM) is a leading noncommunicable disease which affects more than 100 million people worldwide and is considered as one of the fine leading diseases which causes death in the world [26]. Type-2 diabetes mellitus is a chronic metabolic disorder that results from defects in both insulin secretion and insulin action. Management of type-2 diabetes by conventional therapy involves the inhibition of degradation of dietary starch by glucosidases such as *α*-amylase and *α*-glucosidase [27]. The pathogenesis of type-2 diabetes involves progressive development in insulin resistance associated with a defect in insulin secretion, leading to overt hyperglycemia. However, compounds which improve insulin sensitivity and glucose intolerance are somewhat limited warranting the discovery and characterization of novel molecules targeting various pathways involved in the pathogenesis of type-2 diabetes [28]. Currently, there are few drugs that are able to counteract the development of the associated pathologies. Therefore, the need to search for new drug candidates in this field appears to be critical. Aromatic amines and thiazolidinones nuclei would produce new compounds with significant antidiabetic properties [29].

In our research group, we have already reported larvicidal activity of 7-chloro-3,4-dihydro-9-phenylacridin-1(2*H*)-one and (*E*)-7-chloro-3,4-dihyro-phenyl-2-[(pyridin-2-yl)methylene]acridin-1(2*H*)-one [30]. In continuation of our research work on acridine moiety, presently we focus on the synthesis of 10-chloro-4-(2-chlorophenyl)-12-phenyl-5,6-dihydropyrimido[4,5-*a*]acridin-2-amine,** 3a**–**3f** derivatives. All the synthesized dihydropyrimido[4,5-*a*]acridin-2-amines analogues,** 3a**–**3f,** were evaluated for docking and* in vitro* antidiabetic activity.

## 2. Materials and Methods

### 2.1. Chemistry

Melting points were determined by open capillary method and are corrected with standard benzoic acid. All solvents were distilled and dried prior to use. TLC was performed on silica gel G and the spots were exposed to iodine vapour for visualization. A mixture of petroleum ether and ethyl acetate was used as an eluent at different ratio. Column chromatography was performed by using silica gel (60–120 mesh). ^1^H NMR and ^13^C NMR spectra were recorded in CDCl_3_ on a Bruker advance 400 MHz instrument. Chemical shifts are reported in ppm using TMS as the internal standard. IR spectra were obtained on a Perkin-Elmer spectrum RXI FT-IR spectrometer (400–4000 cm^−1^; resolution: 1 cm^−1^) using KBr pellets. Molecular mass was determined using ESI-MS THERMO FLEET spectometer.

### 2.2. Biological Assays

The dialysis membrane, 1,4-*α*-D glucan-glucanohydrolase, *α*-amylase, *α*-glucosidase, P-nitrophenyl-*α*-D-glucopyranoside, and acarbose were purchased from Himedia Laboratories, Mumbai, India. All other chemicals and reagents were AR grade purchased locally.

### 2.3. General Synthesis of 10-Chloro-4,12-diphenyl-5,6-dihydropyrimido[4,5-*a*]acridin-2-amine, **3a–3f**

**Scheme 1:**
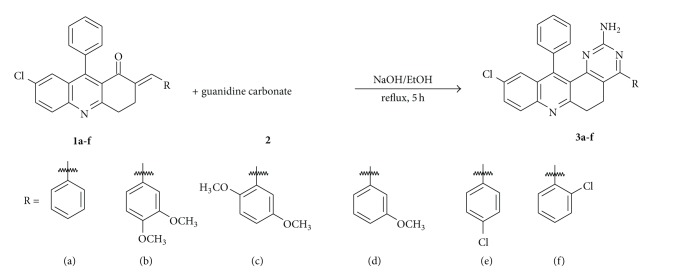
Synthesis of 10-chloro-4,12-diphenyl-5,6-dihydropyrimido[4,5-*a*]acridin-2-amines,** 3a**–**3f**.

10-Chloro-4,12-diphenyl-5,6-dihydropyrimido[4,5-*a*]acridin-2-amines,** 3a**–**3f,** were synthesized* via*., (*E*)-2-benzylidene-7-choloro-3,4-dihydro-9-phenylacridin-1(2*H*)-ones,** 1a**–**1f**, (3,9511 g, 0.01 mol) were mixed with guanidine carbonate** 2** (0.9008 g, 0.01 mol) and 10 mL of 10% alc. NaOH (1 g in 10 mL ethanol) and then heated under reflux condition for 5 h. After the completion of the reaction, reaction mixture was cooled and poured into crushed ice. The crude product was separated by column chromatography using 10–15% of ethyl acetate and pet ether solvent to get the target compounds,** 3a**–**3f**. The synthetic scheme was presented in Scheme 1 and physical data of all synthesized derivatives were summarized in Table 1.

Spectral data of the synthesized compounds are described below.

*10-Chloro-4,12-diphenyl-5,6-dihydropyrimido[4,5-a]acridin-2-amine* **(3a)**. Yellow solid. Yield 70%. mp. 194–196°C. FT-IR (KBr) *ν*_max⁡_ (cm^−1^): 3450.30 (–NH_2_), ^1^H NMR (400 MHz, CDCl_3_): *δ* (ppm), 2.99 (s, 2H, –CH_2_), 3.18 (s, 2H, –CH_2_), 4.43 (s, 2H, –NH_2_), 7.25 (m, 6H), 7.46 (d, 2H), 7.53 (m, 2H), 7.60–7.65 (m, 2H), 7.99–8.02 (d, *J* = 8.8 Hz, 1H). ^13^C NMR (400 MHz, CDCl_3_): *δ* (ppm), 23.8, 33.8, 118.5, 125.0, 126.2, 127.3, 127.9, 128.3, 128.7, 128.8, 129.2, 129.3, 130.2, 131.1, 132.1, 137.8, 137.9, 146.1, 146.9, 160.4, 160.7, 160.9, 165.5, EI-MS* m/z* 435.30 [M+1].

*10-Chloro-4-(3,4-dimethoxyphenyl)-12-phenyl-5,6-dihydropyrimido[4,5-a]acridin-2-amine* **(3b)**. Yellow solid. Yield 81%. mp. 218–220°C. FT-IR (KBr) *ν*_max⁡_ (cm^−1^): 3446.79 (–NH_2_), 2931.80–2958.80 (–OCH_3_). ^1^H NMR (400 MHz, CDCl_3_): *δ* (ppm), 3.03–3.06 (m, 2H, –CH_2_), 3.18–3.21 (m, 2H, –CH_2_), 3.93-3.94 (d, *J* = 3.2 Hz, 6H, 2-OCH_3_) 4.34 (s, 2H, –NH_2_), 6.93–6.95 (d, *J* = 8 Hz, 1H), 7.11 (d, *J* = 2 Hz, 1H), 7.13 (d, *J* = 2 Hz, 1H), 7.15 (d, *J* = 2 Hz, 1H), 7.23–7.26 (m, 2H), 7.44–7.50 (m, 2H), 7.61 (d, *J* = 2.4 Hz, 1H), 7.64 (d, *J* = 2.4 Hz, 1H), 7.66 (d, *J* = 2.4 Hz, 1H). ^13^C NMR (400 MHz, CDCl_3_): *δ* (ppm), 23.9, 32.8, 55.9, 110.9, 117.2, 117.9, 119.3, 120.2, 120.6, 125.3, 127.5, 127.9, 128.1, 128.4, 128.9, 129.0, 130.1, 130.3, 132.3, 135.0, 138.5, 140.3, 140.7, 144.9, 148.6, 149.3, 157.8, 158.4. EI-MS* m/z* 495.52 [M+1].

*10-Chloro-4-(2,5-dimethoxyphenyl)-12-phenyl-5,6-dihydropyrimido[4,5-a]acridin-2-amine* **(3c)**. White solid. Yield 75%. mp. 212–214°C. FT-IR (KBr) *ν*_max⁡_ (cm^−1^): 3485.37 (–NH_2_), 2931.80–2953.02 (–OCH_3_). ^1^H NMR (400 MHz, CDCl_3_): *δ* (ppm), 2.64 (d, *J* = 6.8 Hz, 1H, –CH_2_), 2.87 (d, *J* = 3.2 Hz, 1H, –CH_2_), 3.14–3.22 (dd, *J* = 6.8 Hz, *J* = 17.2 Hz, 2H, –CH_2_), 3.75–3.79 (d, *J* = 18.4 Hz, 6H, 2-OCH_3_), 4.30 (s, 2H, –NH_2_), 6.89–6.97 (m, 3H), 7.31-7.32 (d, *J* = 4.8 Mz, 1H), 7.46 (m, 3H), 7.57-7.58 (d, *J* = 2.4 Mz, 1H), 7.62-7.63 (d, *J* = 2.4 Mz, 1H), 7.65 (d, *J* = 2.4 Hz, 1H), 7.99–8.01 (d, *J* = 8.8 Hz, 1H). ^13^C NMR (400 MHz, CDCl_3_): *δ* (ppm), 23.0, 33.6, 55.8, 56.0, 112.1, 115.4, 115.8, 120.5, 124.9, 126.2, 127.1, 127.8, 128.8, 130.1, 131.0, 131.9, 138.0, 146.0, 146.9, 150.6, 153.7, 159.4, 160.4, 161.2, 163.7. EI-MS* m/z* 495.35 [M+1].

*10-Chloro-4-(3-methoxyphenyl)-12-phenyl-5,6-dihydropyrimido[4,5-a]acridin-2-amine* **(3d)**. Yellow solid. Yield 69%. mp. 168-170°C. FT-IR (KBr) *ν*_max⁡_ (cm^−1^): 3475.73 (–NH_2_), 2924.73–2960.23 (–OCH_3_). ^1^H NMR (400 MHz, CDCl_3_): *δ* (ppm), 2.39-2.40 (m, 2H, –CH_2_), 2.71–2.83 (m, 2H, –CH_2_), 3.82 (s, 3H, –OCH_3_), 4.83 (s, 2H, –NH_2_), 6.91 (d, *J* = 1.2 Hz, 1H), 6.92-6.93 (s, 1H), 6.97–6.99 (d, *J* = 7.2 Hz, 1H), 7.02–7.04 (d, *J* = 8.4 Hz, 1H), 7.12–7.14 (m, 1H), 7.15 (m, 1H), 7.20–7.22 (m, 2H), 7.24 (m, 2H), 7.31–7.33 (d, *J* = 8.4 Hz, 1H), 7.34–7.36 (d, *J* = 7.2 Hz, 1H). ^13^C NMR (400 MHz, CDCl_3_): *δ* (ppm), 23.7, 33.8, 55.3, 114.1, 115.0, 118.5, 121.1, 124.9, 126.2, 127.2, 127.9, 128.7, 129.3, 129.4, 130.2, 131.1, 132.0, 137.8, 139.3, 146.1, 146.8, 159.5, 160.4, 160.6, 160.9, 165.4 EI-MS* m/z* 467.31 [M+3].

*10-Chloro-4-(4-chlorophenyl)-12-phenyl-5,6-dihydropyrimido[4,5-a]acridin-2-amine* **(3e)**. White solid. Yield 71%. mp. 210-212°C. FT-IR (KBr) *ν*_max⁡_ (cm^−1^): 3495.01 (–NH_2_), ^1^H NMR (400 MHz, CDCl_3_): *δ* (ppm), 2.97–3.00 (m, 2H, –CH_2_), 3.18–3.21 (m, 2H, –CH_2_), 4.35 (s, 2H –NH_2_), 7.23–7.26 (m, 2H), 7.43–7.51 (m, 7H), 7.60 (d, *J* = 2 Hz, 1H), 7.64-7.65 (d, *J* = 2.4 Hz, 1H), 7.66-7.67 (d, *J* = 2.4 Hz, 1H). ^13^C NMR (400 MHz, CDCl_3_): *δ* (ppm), 23.8, 33.7, 118.4, 124.8, 126.2, 127.3, 127.9, 128.6, 128.7, 129.3, 130.2, 131.2, 132.1, 135.4, 136.3, 137.7, 146.1, 147.0, 160.4, 160.7, 160.9, 164.3. EI-MS* m/z* 470.03 [M+1].

*10-Chloro-4-(2-chlorophenyl)-12-phenyl-5,6-dihydropyrimido[4,5-a]acridin-2-amine* **(3f)**. Light yellow. Yield 69%. mp. 238-240°C. FT-IR (KBr) *ν*_max⁡_ (cm^−1^): 3481.51 (–NH_2_), ^1^H NMR (400 MHz, CDCl_3_): *δ* (ppm), 3.12–3.16 (m, 4H, 2-CH_2_), 5.72 (s, 2H –NH_2_), 7.26-7.27 (d, *J* = 7.2 Hz, 2H), 7.37 (s, 1H), 7.41–7.44 (m, 1H). 7.46–7.51 (m, 5H), 7.56–7.58 (d, *J* = 7.6 Hz, 1H), 7.79–7.81 (dd, *J* = 2 Hz, *J* = 2 Hz, 1H), 8.04–8.06 (d, *J* = 8.8 Mz, 1H). ^13^C NMR (400 MHz, CDCl_3_): *δ* (ppm), 23.8, 33.7, 118.4, 124.8, 126.2, 127.3, 127.9, 128.6, 128.7, 129.3, 130.2, 131.2, 132.1, 135.4, 136.3, 137.7, 146.1, 147.0, 160.4, 160.7, 160.9, 164.3. EI-MS* m/z* 469.23 [M^+^].

### 2.4. Single Crystal X-Ray Diffraction (XRD) Analysis

**Figure 2:**
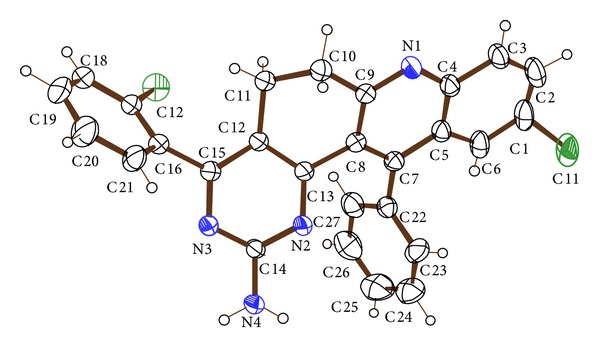
ORTEP diagram of compound** 3f**.

**Figure 3:**
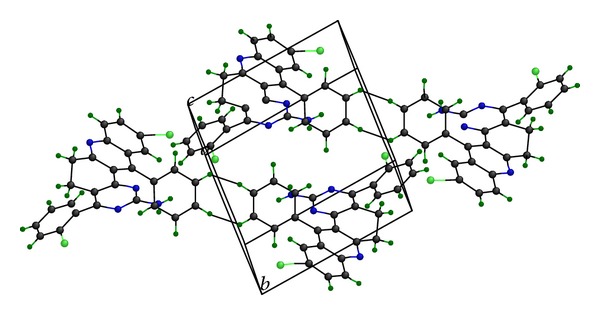
The packing of the molecules in the unit cell, viewed down the a-axis with the hydrogen bond geometry of compound** 3f**.

Crystals suitable for X-ray analysis were obtained by slow evaporation of a solution of the 10-chloro-4-(2-chlorophenyl)-12-phenyl-5,6-dihydropyrimido[4,5-*a*]acridin-2-amine,** 3f**, in ethyl acetate. The measurements were made on Enraf Nonius CAD_4_-MV_31_ single crystal X-ray diffractometer. The diagrams and calculations have been performed using SAINT (APEX II) for frame integration, SHELXTL for structure solution and refinement software programs. The structure was refined using the full-matrix least squares procedures on F^2^ with anisotropic thermal parameters for all nonhydrogen atoms. The crystal data and details concerning data collection and structure refinement of compound,** 3f**, single crystal are summarized in Table 2. As we can see compound** 3f** crystallizes in triclinic space group, P-1. The single crystal structure and atomic numbering chosen for compound** 3f** are demonstrated in Figure 2. Selected bond lengths and bond angles are compiled in Table 3. It can be observed that the bond lengths of all kind atoms on the whole molecule are absorbed. The typical C–C single (1.55 Å) and C=C double (1.38 Å) bonds, C–N typical single (1.35 Å) and C=N double (1.33 Å) bonds. This means that the carbon-carbon bond and the carbon-nitrogen bond have double bond character and contribute to form conjugated system. Furthermore, carbon attached with strong electronegative atom chlorine bond length is C–Cl (1.68 A), in case all C–H (0.97 A) and C=H (0.93 A) are absorbed. The primary amine hydrogen bond length is N–H (0.86 A), respectively (Table 3). Concerning inspection of the torsion angles, the** 3f** molecule has nearly planar core.  Figure 3 illustrates a representative view of compound** 3f** crystal packing structure. The two molecules of compound** 3f** are packed in face-to-face arrangement due to the intermolecular hydrogen bonds. The crystal packing structure demonstrates the existence of two intermolecular hydrogen bonds. Interesting result were absorbed from crystal packing structure is the intermolecular hydrogen bonding occurs at C(23)–H(23) and C(24)–H(24) of one molecule to another neighbouring molecule of C(23)–H(23) and C(24)–H(24), not in the case of primary amine containing two hydrogen N(4)–H(A) and N(4)–H(B) Figure 3.

### 2.5. Glucose Diffusion Inhibitory Test

*Sample Preparation*. Four different concentrations (100, 200, 300, and 400 *μ*g/mL) of samples were prepared. 1 mL of the sample was placed in a dialysis membrane (12000 MW, Himedia laboratories, Mumbai) along with a glucose solution (0.22 mM in 0.15 M NaCl). Then it was tied at both ends and immersed in a beaker containing 40 mL of 0.15 M NaCl and 10 mL of distilled water. The control contained 1 mL of 0.15 M NaCl containing 0.22 mM glucose solution and 1 mL of distilled water. The beaker was then placed in an orbital shaker. The external solution was monitored every half an hour. Three replications of this test were done for 3 hrs [31, 32].

### 2.6. Inhibition Assay for *α*-Amylase Activity

Four different concentrations (100, 200, 300, and 400 *μ*g/mL) of samples and standard drug acarbose were prepared and made up to 1 mL with DMSO. A total of 500 *μ*L of sample and 500 *μ*L of 0.02 M sodium phosphate buffer (pH 6.9 with 0.006 M NaCl) containing *α*-amylase solution (0.5 mg/mL) were incubated for 10 minutes, at 25°C. After preincubation, 500 *μ*L of 1% starch solution in 0.02 M sodium phosphate buffer (pH 6.9 with 0.006 M NaCl) was added to each tube. This reaction mixture was then incubated for 10 minutes at 25°C. 1 mL of DNSA colour reagent was added to stop the reaction. These test tubes were then incubated in a boiling water bath for 5 minutes and cooled to room temperature. Finally this reaction mixture was again diluted by adding 10 mL of distilled water. % of inhibition by *α*-amylase can be calculated by using the following formula. Absorbance was measured at 540 nm [32, 33]:

(1) $$\begin{array}{c} \%\;\text{inhibition}=\frac{A_{540}\;\text{control}-A_{540}\;\text{sample}}{A_{540}\;\text{control}}\times 100. \end{array}$$

Triplicates were done for each sample at different concentrations.

### 2.7. Inhibition Assay for *α*-Glucosidase Activity

Various concentrations of samples and standard drug acarbose were prepared. *α*-Glucosidase (0.075 units) was premixed with sample. 3 mM p-nitrophenyl glucopyranoside used as a substrate was added to the reaction mixture to start the reaction [34]. The reaction was incubated at 37°C for 30 min and stopped by adding 2 mL of Na_2_CO_3_. The *α*-glucosidase activity was measured by p-nitrophenol release from PNPG at 400 nm. % of inhibition can be calculated by using (1).

Triplicates are done for each sample at different concentrations [31–33].

### 2.8. Statistical Analysis

Statistical analysis was performed using one-way analysis of variance (ANOVA). Results are expressed as mean ± SD and *n* = 3.

## 3. Results and Discussion

### 3.1. Glucose Diffusion Inhibitory Test

**Figure 4:**
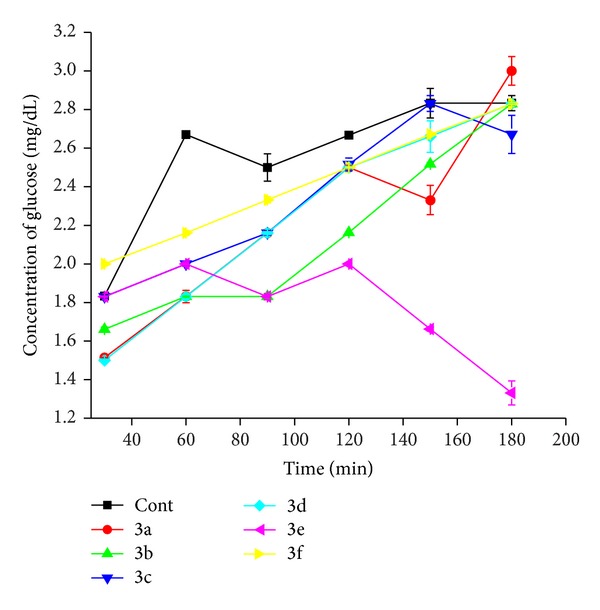
Concentration of glucose (mg/dL).

**Figure 5:**
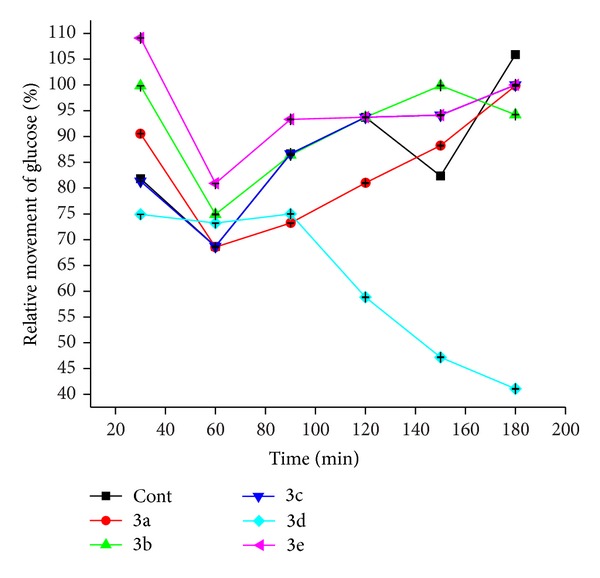
% of relative movement in glucose diffusion inhibitory assay.

The results are summarized in Tables 4 and 5. The diffused glucose concentration is given in Table 4 and % of relative movement is given in Table 5. The movement of glucose from inside of membrane to external solution monitored and compared in Figures 4 and 5. Among the six samples only the** 3e** retains the glucose and shows minimum % of relative movement 41.06% over 180 minutes. All other samples show the highest % of relative movement in glucose diffusion from 30 to 180 minutes.

#### 3.1.1. *α*-Amylase Inhibitory Assay

The results are given in Table 6. All samples show gradual increase in inhibition, where the sample concentration increased from 100 to 400 *μ*g/mL. Sample** 3e** shows maximum inhibition of 57% and sample** 3d** shows 23.55% of inhibition. All other samples show varying less significant inhibition.

#### 3.1.2. *α*-Glucosidase Inhibition Assay

The results are shown in Table 7. Among the six samples,** 3e** shows maximum inhibitory activity of 60.25% at higher concentration (400 *μ*g/mL). Sample** 3d** shows 23.01% of inhibition. All the other samples are less significant.

### 3.2. Molecular Docking Studies

The synthesized molecules** 3a**–**3f** were constructed using* VEGA ZZ* molecular modeling package [35]. The obtained structures were then geometrically optimized using AM1 Hamiltonian in MOPAC software package [36]. The X-ray structure of pig *α*-pancreatic *α*-amylase (PDB 3L2M) and N-terminal human maltase-glucoamylase with* Casuarina* (PDB 3CTT) [37], obtained from Brookhaven Protein Data Bank, were used for docking calculations. Docking calculations were performed with AutoDock 4.0 [38].

The crystal structures were refined by removing water molecules and repeating coordinates. Hydrogen atoms were added and charges were assigned to the protein atoms using Kollman united atoms force field by using AutoDockTools-1.5.6. For docking calculations, Gasteiger partial atomic charges were added to the synthesized structures and all possible flexible torsion angles of the ligand were defined by using AUTOTORS. The structures were saved in a PDBQT format for AutoDock calculations.

AutoDock requires precalculated* grid maps*, one for each atom type present in the structure being docked. The auxiliary program AutoGrid generated the grid maps. Lennard-Jones parameters 12-10 and 12-6, implemented with the program, were used for modeling H-bonds and van der Waals interactions, respectively. The Lamarckian genetic algorithm method was applied for docking calculations using default parameters. AutoDock uses a semiempirical free energy force field to evaluate conformations during docking simulations. The optimized orientations represent possible binding modes of the ligand within the site:

(2) $$\begin{array}{c} \Delta G=\Delta G_{\text{vdw}}+\Delta G_{\text{hbond}}+\Delta G_{\text{elec}}+\Delta G_{\text{tor}}+\Delta G_{\text{desolv}}. \end{array}$$

The first three terms are van der Waals, hydrogen bonding, and electrostatics, respectively. The term Δ*G*_tor_ is for rotation and translation and Δ*G*_desolv_ is for desolvation upon binding and the hydrophobic effect.

After docking, the 50 solutions were clustered into groups with RMS deviations lower than 1.0 Å. The clusters were ranked by the lowest energy representative of each cluster.

#### 3.2.1. Molecular Docking Study on *α*-Amylase

The binding site of the structures was not identified because of the absence of the crystal structure of the ligand, and a blind docking was performed for all the structures** 3a**–**3f** with the protein structure 3L2 M [39]. Two interacting binding sites were identified [37], one near the entrance of the central beta-barrel of the enzyme and the other near the N-terminal of the protein [39]. The docked structures showed binding energy in the range of −4.2 to −4.8 Kcal/mol. It was observed that the structure** 3e** exhibits high binding energy of −4.8 Kcal/mol and the structure** 3d** exhibits binding energy of −4.5 Kcal/mol, respectively. The binding energies (Δ*G*_BE_) and intermolecular energies (Δ*G*_intermol_) of the structures** 3d** and** 3e** obtained for *α*-amylase are given in Table 8. When comparing** 3d** and** 3e** structures,** 3e** exhibits high binding energy. The structure** 3e** shows interaction with the residues* Trp58*,* Trp59*,* Tyr62*,* Asp197*, and* Asp300* present in the binding site of *α*-amylase. The amino acids* Trp58* and* Trp59* were the key residues interacting with all the structures. The docking studies revealed that the van der Waals, electrostatic, and desolvation energies play a key role in binding. It was observed that the Pyrimido ring was fitted well with binding site, via hydrogen bonds through the hydrogen atom of the amino group of** 3e** with carboxylate group of* Asp300*. The hydrophobic interactions were formed by* Trp58*,* Trp59*,* Gln63*,* Val163*,* Asp300*,* His305*, and* Asp356* in** 3e**.

#### 3.2.2. Molecular Docking Study on *α*-Glucosidase

All the binding modes of the structures** 3a**–**3f** were explored using the docking calculation in AutoDock. The binding site of the structures was identified using the crystal structure of* Casuarina*. The docked structures showed binding energy in the range of −6.3 to −6.6 Kcal/mol. The binding energies (Δ*G*_BE_) and intermolecular energies (Δ*G*_intermol_) of the structures** 3d** and** 3e** obtained for *α*-glucosidase are given in Table 8. The structure** 3e** showed high binding energy when compared to** 3d**. Based on docking calculations, the synthesized compounds** 3d** and** 3e** show better inhibition of *α*-glucosidase compared to *α*-amylase which is in a good agreement with* in vitro* studies. The following key residues* Asp203*,* Tyr299*,* Trp406*,* Asp443*,* Met444*,* Phe450*,* Lys480*,* Asp542*,* Phe575*, and* Gln603* present in the binding site of *α*-glucosidase show noncovalent interactions with** 3e**. It was observed that the Pyrimido ring of** 3e** formed hydrogen bonds through the hydrogen atom of the amino group with carboxylate group of* Asp203* at a distance of 1.9 Å. The residues* Tyr299*,* Trp406*,* Asp443*,* Met444*,* Phe450*,* Lys480*,* Asp542*,* Phe575,* and* Gln603* formed hydrophobic interactions with** 3e**.

In diabetes mellitus, control of postprandial plasma glucose level is critical in the early treatment [34]. Inhibition of enzymes involved in the metabolism of carbohydrates are one of the therapeutic approaches for reducing postprandial hyperglycemia. The inhibition by natural products is more safe than synthetic drugs.

*α*-Amylase and *α*-glucosidase are significant enzymes which cleave carbohydrates responsible for absorption of glucose in the blood stream. For this reason, synthetic drug such as acarbose, miglitol, and voglibose are some inhibitors which inhibit *α*-amylase and *α*-glucosidase [40]. However, these agents have their limitations, because they are nonspecific and produce serious side effects such as GI tract inflammation and to elevate diabetic complications [41].

## 4. Conclusion

The present study revealed that we have successfully achieved our target title compound 10-chloro-4,12-diphenyl-5,6-dihydropyrimido[4,5-*a*]acridin-2-amine derivatives. All compounds were confirmed by suiTable experimental and spectroscopic techniques. Synthesized derivatives (**3a**–**3f**) were evaluated* in vitroα*-amylase and *α*-glucosidase inhibitory activity and glucose diffusion test of samples. Among all other derivatives, compounds 10-chloro-4-(4-chlorophenyl)-12-phenyl-5,6-dihydropyrimido[4,5-*a*]acridin-2-amine,** 3e**, and 10-chloro-4-(3-methoxyphenyl)-12-phenyl-5,6-dihydropyrimido[4,5-*a*]acridin-2-amine,** 3d**, show good inhibitory activity for *α*-amylase and *α*-glucosidase with the values of 57.96, 60.27, 23.55, and 23.01% for compounds** 3e** and** 3d**, respectively. The docking studies are in good agreement with the* in vitro* studies. The docking calculations showed that van der Waals, electrostatic, and desolvation energies play a key role in binding. These factors are considered for designing new inhibitors for *α*-amylase and *α*-glucosidase.

## Supplementary Material

CCDC 988641 contains the supplementary crystallographic data for this paper. These data can be obtained free of charge from the Cambridge Crystallographic Data Centre via https://www.ccdc.cam.ac.uk/data-request/cif or from the Cambridge Crystallographic Data Centre, 12 Union Road, Cambridge CB2 1EZ, UK; fax: +44 1223 336033; or e-mail: deposit@ccdc.cam.ac.uk.

## Figures and Tables

**Table 1:** Summary of synthesized 2-aminopyrimidine derivatives (**3a–f**) via Scheme 1.

S. no.	Compounds	R	M.P. (°C)	Yield%
1	**3a**	-H	194–196	70
2	**3b**	-3,4-OCH_3_	218–220	81
3	**3c**	-2,5-OCH_3_	212–214	75
4	**3d**	-2-OCH_3_	168–170	69
5	**3e**	-4-Cl	210–212	71
6	**3f**	-2-Cl	238–240	69

**Table 2:** The crystallographic data and structure refinement parameters of compound **3f**.

Empirical formula	C_27_ H_18 _Cl_2_ N_4_
Formula weight	469.35
Temperature	293(2) K
Wavelength	0.71073 Å
Crystal system, space group	Triclinic, P-1
Unit cell dimensions	*a* = 9.9421(5) Å, *α* = 67.248(2)°.
	*b* = 10.9553(5) A, *β* = 78.530(2)°.
	*c* = 11.5126(5) A, *γ* = 89.875(2)°.
Volume	1129.39(9) A^3^
*Z*	2
Calculated density	1.380 Mg/m^3^
Absorption coefficient	0.311 mm^−1^
*F*(000)	484
Crystal size	0.35 × 0.30 × 0.25 mm
Theta range for data collection	1.96 to 25.00°.
Limiting indices	−11 ≤ *h* ≤ 11, −12 ≤ *k* ≤ 12, −13 ≤ *l* ≤ 13
Reflections collected/unique	19503/3929 [*R*(int) = 0.0276]
Completeness to theta = 25.00	98.8%
Absorption correction	Semiempirical from equivalents
Max. and min. transmission	0.9635 and 0.8623
Refinement method	Full-matrix least squares on *F* ^2^
Data/restraints/parameters	3929/6/308
Goodness-of-fit on *F* ^2^	1.200
Final *R* indices [*I* > 2 sigma(*I*)]	*R*_1_ = 0.0569, *wR*_2_ = 0.1944
*R* indices (all data)	*R*_1_ = 0.0712, *wR*_2_ = 0.2074
Largest diff. peak and hole	0.383 and −0.385 *e*·A^−3^

**Table 3:** Some important bond lengths (A) and bond angles (°) of compound **3f**.

Bond	Bond length	Bond	Bond angle
C(1)–C(6)	1.371(6)	N(1)–C(4)–C(5)	123.2(3)
C(2)–H(2)	0.9300	N(1)–C(4)–C(3)	117.1(4)
C(11)–H(11A)	0.9700	N(1)–C(9)–C(8)	124.1(4)
C(11)–H(11A)	0.9700	N(1)–C(9)–C(10)	116.4(3)
C(11)–H(11B)	0.9700	C(8)–C(9)–C(10)	119.4(3)
C(11)–H(11B)	0.9700	C(9)–C(10)–H(10A)	109.5
C(13)–N(2)	1.330(4)	C(11)–C(10)–H(10A)	109.5
C(14)–N(2)	1.332(4)	C(12)–C(11)–C(10)	109.0(3)
C(14)–N(3)	1.344(4)	H(11A)–C(11)–H(11B)	108.3
C(14)–N(4)	1.354(4)	C(13)–C(12)–C(15)	115.8(3)
C(15)–N(3)	1.339(4)	C(15)–C(12)–C(11)	124.3(3)
N(4)–H(4A)	0.8600	N(2)–C(13)–C(12)	123.0(3)
N(4)–H(4B)	0.8600	N(2)–C(13)–C(8)	117.8(3)
C(4)–N(1)	1.361(6)	C(12)–C(13)–C(8)	119.1(3)
C(9)–N(1)	1.317(5)	N(2)–C(14)–N(3)	126.1(3)
C(9)–C(10)	1.496(6)	N(2)–C(14)–N(4)	117.0(3)
C(10)–C(11)	1.525(6)	N(3)–C(14)–N(4)	116.9(3)
C(11)–C(12)	1.508(5)	N(3)–C(15)–C(12)	122.4(3)
C(26)–H(26)	0.9300	N(3)–C(15)–C(16)	116.7(3)
C(27)–H(27)	0.9300	C(13)–N(2)–C(14)	116.4(3)
C(9)–N(1)	1.317(5)	C(15)–N(3)–C(14)	116.2(3)
C(1)–Cl(1′)	1.681(11)	C(14)–N(4)–H(4B)	120.0
C(1)–Cl(1)	1.99(4)	H(4A)–N(4)–H(4B)	120.0
C(17)–Cl(2)	1.732(4)		

**Table 4:** Release of glucose through dialysis membrane to external solution (mg/dL).

Time (min)	Control	**3a**	**3b**	**3c**	**3d**	**3e**	**3f**
30	1.833 ± 0.00	1.515 ± 0.01	1.661 ± 0.00	1.830 ± 0.00	1.5 ± 0.00	1.830 ± 0.00	2 ± 0.00
60	2.670 ± 0.03	1.831 ± 0.00	1.831 ± 0.00	2 ± 0.01	1.83 ± 0.01	2 ± 0.01	2.16 ± 0.00
90	2.500 ± 0.00	2.161 ± 0.01	1.831 ± 0.01	2.160 ± 0.00	2.16 ± 0.01	1.831 ± 0.01	2.332 ± 0.07
120	2.667 ± 0.01	2.5 ± 0.00	2.162 ± 0.03	2.515 ± 0.00	2.5 ± 0.00	2 ± 0.00	2.5 ± 0.01
150	2.833 ± 0.07	2.331 ± 0.00	2.511 ± 0.04	2.831 ± 0.08	2.66 ± 0.00	1.662 ± 0.00	2.671 ± 0.07
180	2.833 ± 0.07	3 ± 0.00	2.830 ± 0.00	2.671 ± 0.00	2.831 ± 0.06	1.331 ± 0.00	2.83 ± 0.03

Values are mean ± SEM for groups of 3 observations.

**Table 5:** % of relative movement in glucose diffusion inhibitory assay.

Time (min)	**3a**	**3b**	**3c**	**3d**	**3e**	**3f**
30	81.83 ± 0.01	90.56 ± 0.03	99.83 ± 0.00	81.33 ± 0.00	74.90 ± 0.00	109.11 ± 0.01
60	68.66 ± 0.07	68.53 ± 0.00	74.91 ± 0.07	68.66 ± 0.01	73.20 ± 0.02	80.90 ± 0.02
90	86.66 ± 0.05	73.20 ± 0.03	86.40 ± 0.06	86.67 ± 0.02	74.99 ± 0.03	93.33 ± 0.03
120	93.73 ± 0.04	80.99 ± 0.05	93.74 ± 0.05	93.74 ± 0.05	58.83 ± 0.05	93.74 ± 0.07
150	82.36 ± 0.01	88.24 ± 0.06	99.89 ± 0.03	94.13 ± 0.06	47.18 ± 0.07	94.13 ± 0.08
180	105.89 ± 0.02	99.84 ± 0.07	94.24 ± 0.02	100 ± 0.07	41.06 ± 0.06	100 ± 0.06

Values are mean ± SEM for groups of 3 observations.

**Table 6:** % inhibition of *α*-amylase assay.

Concentration (*µ*g/mL)	Acarbose	**3a**	**3b**	**3c**	**3d**	**3e**	**3f**
100	32.01 ± 0.09	7.97 ± 0.01	5.43 ± 0.06	3.26 ± 0.00	18.83 ± 0.02	19.20 ± 0.01	12.23 ± 0.00
200	48.15 ± 0.11	9.41 ± 0.02	5.43 ± 0.02	4.34 ± 0.01	20.65 ± 0.03	21.37 ± 0.00	13.77 ± 0.01
300	70.03 ± 0.25	11.22 ± 0.07	6.52 ± 0.04	5.070 ± 0.08	21.73 ± 0.01	53.62 ± 0.03**	14.85 ± 0.02
400	80.02 ± 0.71	12.31 ± 0.06	6.52 ± 0.04	6.16 ± 0.06	23.55 ± 0.01	57.96 ± 0.04	16.66 ± 0.03

Values are mean ± SEM for groups of 3 observations.

**P < 0.05.

**Table 7:** % inhibition of *α*-glucosidase assay.

Concentration (*µ*g/mL)	Acarbose	**3a**	**3b**	**3c**	**3d**	**3e**	**3f**
100	30.08 ± 0.05	6.02 ± 0.01	6.29 ± 0.00	4.29 ± 0.01	20.91 ± 0.00	21.45 ± 0.01*	14.02 ± 0.02
200	45.02 ± 0.12	8.32 ± 0.01	7.31 ± 0.02	5.02 ± 0.01	21.65 ± 0.01	24.62 ± 0.09	10.77 ± 0.03
300	68.25 ± 0.21	10.12 ± 0.01	8.01 ± 0.03	6.09 ± 0.02	22.71 ± 0.02	56.85 ± 0.01	15.85 ± 0.04
400	78.01 ± 0.07	11.45 ± 0.02	8.25 ± 0.06	9.19 ± 0.02	23.01 ± 0.04	60.25 ± 0.01**	17.71 ± 0.03

Values are mean ± SEM for groups of 3 observations.

*P < 0.01.

**P < 0.05.

**Table 8:** The binding energy (Δ*G*_BE_) and intermolecular energy (Δ*G*_intermol_) of the structures **3d** and **3e **are given. The docking energy and binding energy are reported in Kcal/mol.

Structures	Δ*G*_BE_	Δ*G*_intermol_		Δ*G*_internal_	Δ*G*_tor_
		Δ*G*_vdw_hb_desol_	Δ*G*_elec_		
*α*-Amylase					
**3d**	−4.52	−5.31	−0.40	−1.57	1.19
**3e**	−4.83	−5.35	−0.37	−1.54	0.89

*α*-Glucosidase					
**3d**	−6.51	−6.34	−1.37	−1.44	1.19
**3e**	−6.61	−6.95	−0.56	−1.41	0.89
