# Supplementary figures and images for: In Silico Molecular Docking and In Vitro Antidiabetic Studies of Dihydropyrimido[4,5-a]acridin-2-amines
Source: Biomed Res Int. 2014 Jun 2;2014:971569. doi: 10.1155/2014/971569 (PMC4060768; doi:10.1155/2014/971569)

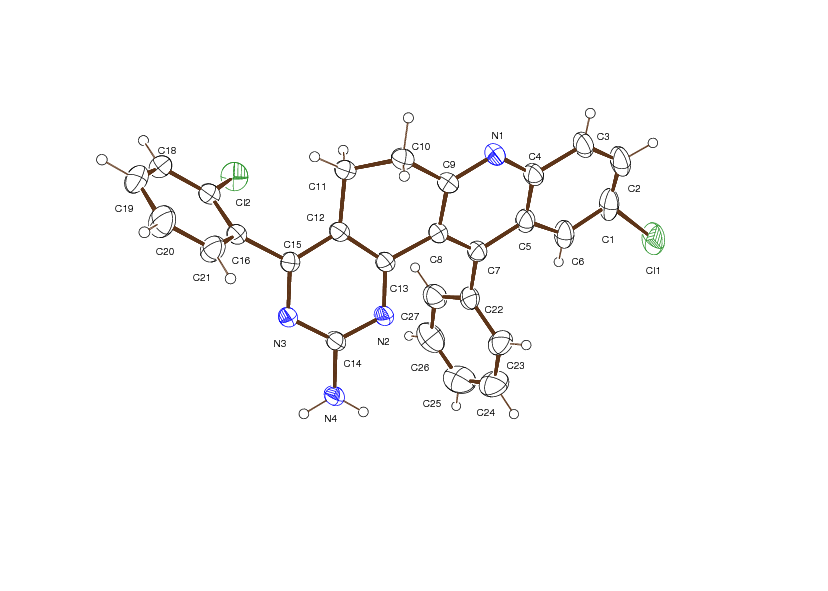

Supplement: Supplementary file 1 — CCDC 988641 contains the supplementary crystallographic data for this paper. These data can be obtained free of charge from the Cambridge Crystallographic Data Centre via https://www.ccdc.cam.ac.uk/data-request/cif or from the Cambridge Crystallographic Data Centre, 12 Union Road, Cambridge CB2 1EZ, UK; fax: +44 1223 336033; or e-mail: deposit@ccdc.cam.ac.uk. [file 971569.f1.zip › 971569.f1/singlecrystalxrddata/ORTEP1.TIF]

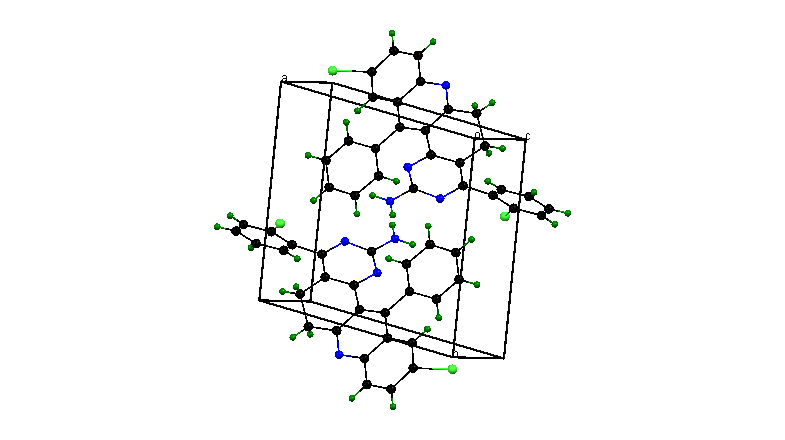

Supplement: Supplementary file 1 — CCDC 988641 contains the supplementary crystallographic data for this paper. These data can be obtained free of charge from the Cambridge Crystallographic Data Centre via https://www.ccdc.cam.ac.uk/data-request/cif or from the Cambridge Crystallographic Data Centre, 12 Union Road, Cambridge CB2 1EZ, UK; fax: +44 1223 336033; or e-mail: deposit@ccdc.cam.ac.uk. [file 971569.f1.zip › 971569.f1/singlecrystalxrddata/PACK UNIT CELL.tif]

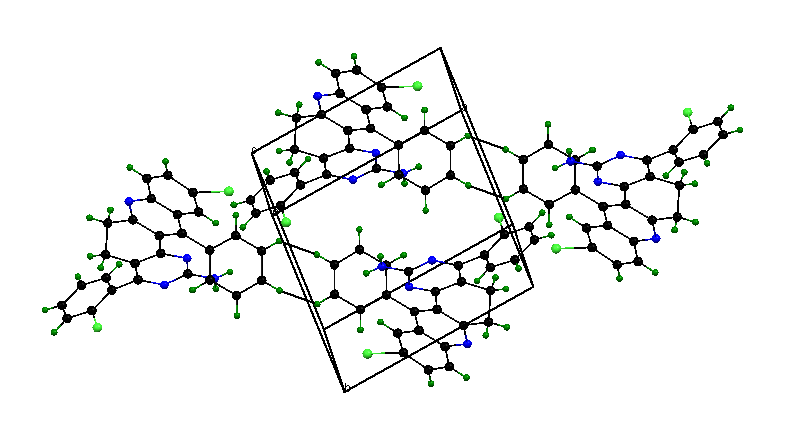

Supplement: Supplementary file 1 — CCDC 988641 contains the supplementary crystallographic data for this paper. These data can be obtained free of charge from the Cambridge Crystallographic Data Centre via https://www.ccdc.cam.ac.uk/data-request/cif or from the Cambridge Crystallographic Data Centre, 12 Union Road, Cambridge CB2 1EZ, UK; fax: +44 1223 336033; or e-mail: deposit@ccdc.cam.ac.uk. [file 971569.f1.zip › 971569.f1/singlecrystalxrddata/PACK.tif]
